# Diagnostic accuracy of computed tomography for differentiating diffuse thyroid disease from normal thyroid parenchyma: A multicenter study

**DOI:** 10.1371/journal.pone.0205507

**Published:** 2018-11-15

**Authors:** Hye Jin Baek, Dong Wook Kim, Yoo Jin Lee, Hye Jung Choo, Hye Shin Ahn, Hyun Kyung Lim, Ji Hwa Ryu

**Affiliations:** 1 Department of Radiology, Gyeongsang National University School of Medicine and Gyeongsang National University Changwon Hospital, Changwon, South Korea; 2 Department of Radiology, Busan Paik Hospital, Inje University College of Medicine, Busan, South Korea; 3 Department of Radiology, Chung-Ang University Hospital, Chung-Ang University College of Medicine, Seoul, South Korea; 4 Department of Radiology, Soonchunhyang University Seoul Hospital, Soonchunhyang University College of Medicine, Seoul, South Korea; 5 Department of Radiology, Haeundae Paik Hospital, Inje University College of Medicine, Busan, South Korea; A.C. Camargo Cancer Center, BRAZIL

## Abstract

This study aimed to assess the diagnostic performance of computed tomography (CT) for differentiating diffuse thyroid disease (DTD) from normal thyroid parenchyma (NTP) using multicenter data. Between January 2016 and June 2016, 229 patients underwent preoperative neck CT and subsequent thyroid surgery at five participating institutions. The neck CT images of each patient were retrospectively reviewed and classified into the following four categories: no DTD, indeterminate, suspicious for DTD, and DTD. The results of the CT image evaluations were compared with the histopathological results to determine the diagnostic accuracy of CT at each institution. According to the histopathological results, there were NTP (n = 151), Hashimoto thyroiditis (n = 24), non-Hashimoto lymphocytic thyroiditis (n = 47), and diffuse hyperplasia (n = 7). The CT categories of the 229 patients were “no DTD” in 89 patients, “indeterminate” in 40 patients, “suspicious for DTD” in 42 patients, and “DTD” in 58 patients. The presence of two or more CT features of DTD, which was classified as “suspicious for DTD” by all radiologists, had the largest area under the receiver-operating characteristic curve (Az = 0.820; 95% confidence interval: 0.764, 0.868), with sensitivity of 85.9% and specificity of 78.2%. However, no statistical significance between readers’ experience and their diagnostic accuracy was found. In conclusion, evaluations of CT images are helpful for differentiating DTD from NTP.

## Introduction

Diffuse thyroid disease (DTD), a major cause of thyroid dysfunction, is classified into autoimmune and non-autoimmune diseases. Two common thyroid autoimmune diseases are Graves’ disease, which is usually associated with hyperthyroidism, and Hashimoto thyroiditis, which is typically associated with hypothyroidism [1]. Previous studies have suggested an association between DTD and thyroid malignancy, although the clinical significance of this relationship is still under debate [2–4]. Therefore, regular monitoring of patients with DTD is performed at many institutions. Cases of symptomatic DTD are easily diagnosed by clinical and serological examinations, such as thyroid autoantibody or thyroid function tests; however, reliable diagnostic tools for detecting asymptomatic or subclinical DTD have not been established [5–8].

Ultrasonography (US) is firstly used for evaluating thyroid disease, whereas computed tomography (CT) is restrictively used for the preoperative tumor and nodal staging in patients with thyroid malignancy. However, neck CT is widely used for evaluating benign and malignant neck lesions, and its scan range include the thyroid gland. Thus, the establishment of specific CT features for detecting asymptomatic or subclinical DTD may be useful for managing patients with asymptomatic or subclinical DTD. Recently, some studies suggest that US and CT of the thyroid gland may be helpful for detecting and managing asymptomatic or subclinical DTD [5–8]. However, the role of imaging-based DTD diagnoses remains controversial despite technological advances and increasing use of ultrasonography and CT in daily clinical practice. Thus, the purpose of this study was to investigate the characteristic CT features and the diagnostic accuracy of CT and for differentiating DTD from normal thyroid parenchyma (NTP) in patients who underwent thyroid surgery, using retrospective image analysis by five radiologists.

## Patients and methods

### Patients

This study follows the principles expressed in the Declaration of Helsinki, and this retrospective study examined patient data collected from five institutions that served as tertiary referral centers. This study was approved by institutional review boards of all participating institutions (Chun-Ang University Hospital, Gyeongsang National University Changwon Hospital, Soonchunhyang University Seoul Hopsital, Haeundae Paik Hospital, and Busan Paik Hospital), and informed consent was waived owing to the retrospective nature of analysis and use of anonymized data. The study design was approved by the Institutional Review Board of Busan Paik Hospital (IRB 16–0269). From January to June 2016, only those patients who met the following criteria at each institution were included: 1) underwent thyroid surgery, 2) underwent neck CT before thyroid surgery, and 3) had available histopathological results for underlying DTD or NTP. Ultimately, a total of 229 patients (age range, 21–80 years; mean age, 46.3 ± 13.1 years) were included in the study.

### Preoperative neck CT

Neck CT (slice thickness, 1–2 mm; reconstruction increment, 2–3 mm; 120–250 mA, 120 kVp; 80–300 mA) was conducted by using a contrast medium and 64-channel multi-detector CT scanners (Brilliance 64, Philips Medical Systems, Cleveland, OH, USA; Discovery CT 750HD SP 64, General Electric Medical Systems, Milwaukee, WI, USA), 128-channel multi-detector CT scanners (LightSpeed, General Electric Medical Systems; SOMATOM Definition AS+, Siemens Healthcare, Forchheim, Germany), or 320-channel multi-detector CT scanners (Aquilion ONE, Toshiba Medical Systems, Otawara, Japan). Non-enhanced axial, contrast-enhanced axial, and contrast-enhanced coronal reformatted CT images were acquired in all patients.

### CT image analysis

Five board-certified radiologists (with 4, 15, 8, 10, and 6 years of experience in neck CT interpretation after obtaining board certification, respectively) retrospectively analyzed the patients’ CT images using a picture-archiving and communication system. Each radiologist investigated the CT features of the patients at their respective institutions. All of the radiologists were blinded to the patients’ ultrasonography or other imaging diagnoses, clinical and serological information, and medication history for DTD. The number of study patients at each institution was 45, 44, 50, 40, and 50 patients.

The following CT features of the thyroid gland were retrospectively investigated: the degree (iso- [normal], decreased, or increased) and pattern (homogeneous or inhomogeneous) of parenchymal attenuation, glandular size (1–2 cm [normal], <1 cm, or >2 cm) and margin (smooth or lobulated), and degree (iso- [normal], decreased, or increased) and pattern (homogeneous or inhomogeneous) of parenchymal enhancement [6, 7, 9]. The Hounsfield unit (HU) values were measured separately in both thyroid lobes by using regions of interest that were placed on non-enhanced and contrast-enhanced CT images, respectively, and then averaged.

**Fig 1:**
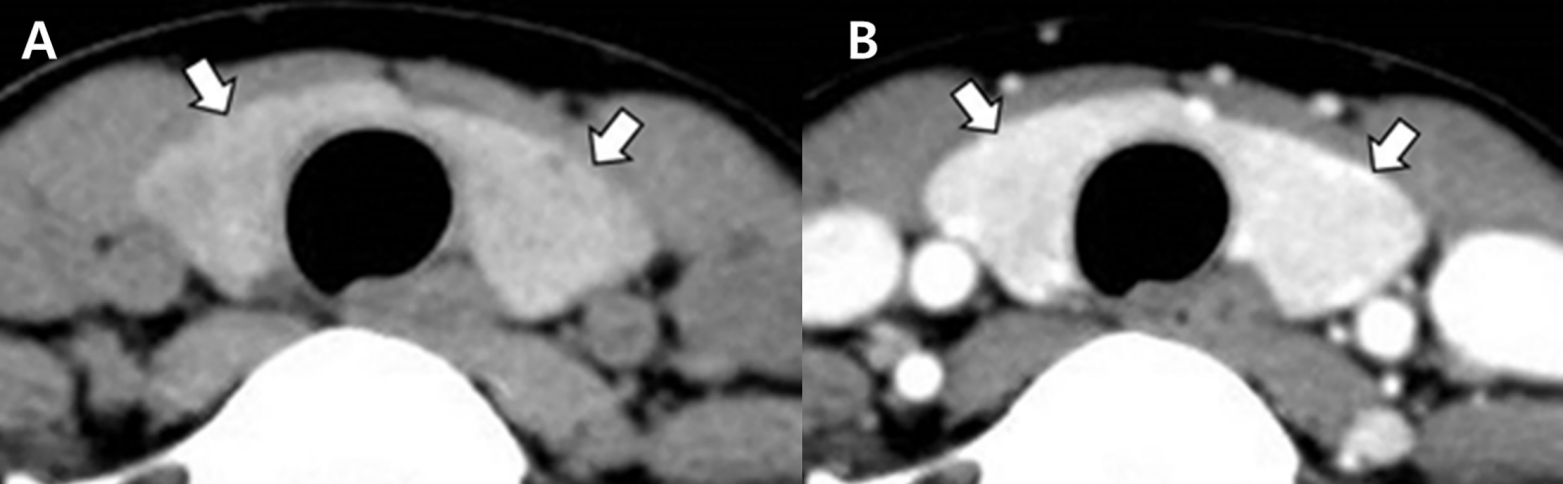
Computed tomography (CT) images in a 37-year-old woman with normal thyroid parenchyma on the histopathological examination that was designated as “no DTD” CT category (papillary thyroid carcinoma in the left lobe). The thyroid gland (arrows) shows iso- and homogeneous attenuation in the non-enhanced CT image (A), normal and homogeneous enhancement in the contrast-enhanced CT image (B), and an anteroposterior diameter of 1–2 cm and a smooth margin in both the non-enhanced and contrast-enhanced CT images.

**Fig 2:**
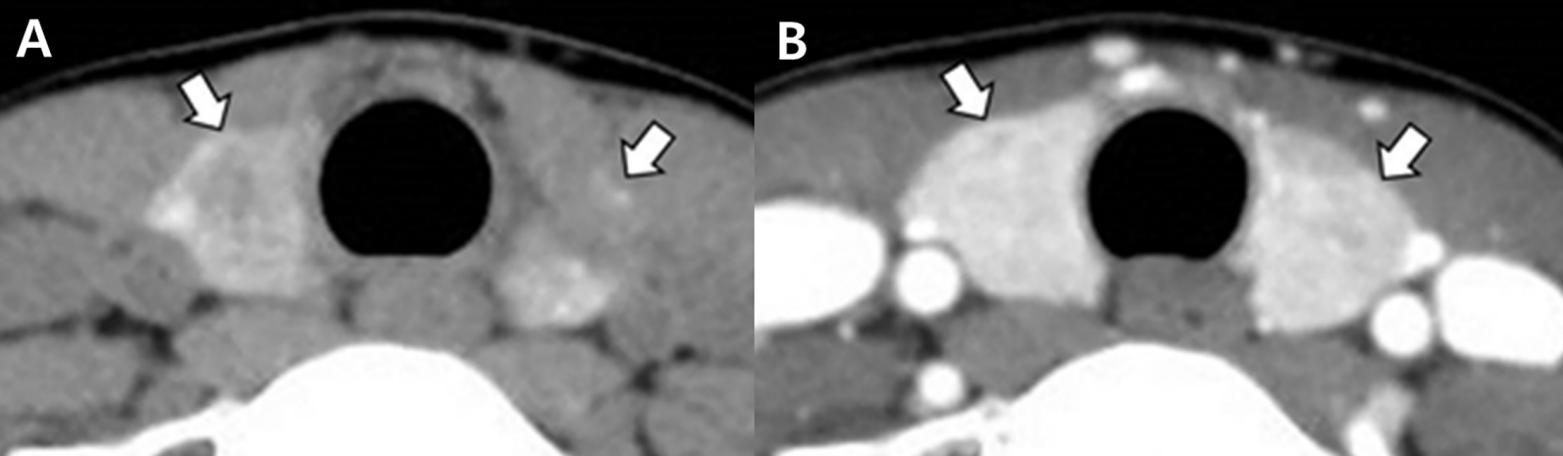
Computed tomography (CT) images in a 24-year-old woman with Hashimoto thyroiditis that was designated as “diffuse thyroid disease” CT category (papillary thyroid carcinoma in the right lobe). The thyroid gland (arrows) shows decreased and inhomogeneous attenuation in the non-enhanced CT image (A), and decreased and inhomogeneous enhancement in the contrast-enhanced CT image (B), but an anteroposterior diameter of 1–2 cm and a smooth margin in both the non-enhanced and contrast-enhanced CT images.

Based on the CT features, the enrolled cases were classified into four categories, as follows: DTD (≥3 abnormal CT features), suspicious for DTD (2 abnormal CT features), indeterminate (1 abnormal CT feature), and no DTD (no abnormal CT feature) (Figs 1 and 2).

### Determination of reference standards according to the histopathological findings

The histopathological analysis of the thyroid gland was performed by board-certified pathologists from each of the affiliated institutions as following [6–9]: Hashimoto thyroiditis (progressive loss of the thyroid follicular cells with replacement by lymphocytes and formation of germinal centers associated with fibrosis), non-Hashimoto lymphocytic thyroiditis (diffuse infiltration of the thyroid gland with lymphocytes and other inflammatory cells but absence of the typical histopathological features of Hashimoto thyroiditis, such as oxyphilic metaplasia, follicular atrophy, and follicular disruption), diffuse hyperplasia (diffuse hypertrophy and hyperplasia of the follicular cells with retention of the lobular architecture and no definite nodule formation), and NTP (when no histopathological evidence of coexisting DTD was found).

### Statistical analysis

To evaluate the differences in the CT features between DTD and NTP, we used independent *t*-tests for continuous variables and Pearson’s chi-squared test or, for small cell values, Fisher’s exact test for categorical variables. The Mantel-Haenszel chi-squared test was also used to evaluate the linear association between the CT category and the incidence of DTD. A receiver operating characteristic (ROC) curve analysis was applied to obtain the optimal HU cutoff value for the thyroid gland on CT images. A cut-off value for each variable was determined by maximizing the sum of the sensitivity and specificity.

Univariate logistic regression analyses were first used to evaluate the predictive power of individual CT features. The CT features with the highest predictive power (P < 0.20, Wald test) were selected and entered into a multivariate logistic regression analysis to determine the optimal logistic regression model for differentiating DTD from NTP. The results of this analysis are presented as odds ratio estimates with corresponding 95% confidence intervals (CI) and *p* values from the Wald test.

An ROC curve analysis was constructed to evaluate the diagnostic accuracy of the best DTD predictor at each institution. The area under the ROC curve (Az) was compared by using the method of DeLong et al. [10]. Kendall’s tau coefficient was also calculated to evaluate the linear correlation between the readers’ experience and the diagnostic accuracy after converting these two variables to ordinal data. All statistical analyses were performed with SPSS version 24.0 and MedCalc version 14.10; statistical significance was set at *p* < 0.05.

## Results

### Patient characteristics

Of the 229 patients, 78 (mean age, 44.7 years; age range, 21–76 years) were subsequently classified as having DTD and 151 (mean age, 47.1 years; age range, 21–80 years) were classified as having NTP. The types of thyroid surgery were hemithyroidectomy (127/229, 55.5%), total thyroidectomy (99/229, 43.2%), and subtotal thyroidectomy (3/229, 1.3%). After thyroid surgery, papillary thyroid carcinoma (207/229, 90.4%), follicular thyroid carcinoma (1/229, 0.4%), medullary thyroid carcinoma (2/229, 0.9%), follicular adenoma (7/229, 3.1%), nodular hyperplasia (9/229, 3.9%), poorly differentiated carcinoma (1/229, 0.4%), and uncontrolled hyperthyroidism with diffuse hyperplasia (2/229, 0.8%) were identified. The histopathological results of the thyroid in the 229 patients were as follows: NTP (151/229, 65.9%), Hashimoto thyroiditis (24/229, 10.5%), non-Hashimoto lymphocytic thyroiditis (47/229, 20.5%), and diffuse hyperplasia (7/229, 3.1%).

### Analyses of the CT features and histopathological results

Comparisons of the CT features between patients with NTP and those with DTD are summarized in Table 1. The degree and pattern of parenchymal attenuation, glandular size, degree and pattern of parenchymal enhancement, thyroid margin, and CT category were significantly different between patients with NTP and those with DTD. However, no significant difference in age was noted between individuals with NTP (mean ± standard deviation [SD], 47.1 ± 12.9) and patients with DTD (44.7 ± 13.5) (*p* = 0.193).

**Table 1:** Comparison of the computed tomography features of normal thyroid parenchyma and diffuse thyroid disease in 229 patients.

Histopathology CT features	Normal thyroid parenchyma	Diffuse thyroid disease	P value
HU on NECT*	107.9 ± 19.4	92.7 ± 25.3	<0.0001
HU on CECT*	209.5 ± 36.3	192.9 ± 35.2	0.001
Degree of attenuation			<0.0001
iso-(normal)	124 (82.1)	33 (42.3)	
decreased	26 (17.2)	45 (57.7)	
increased	1 (0.7)	0	
Pattern of attenuation			<0.0001
homogeneous	111 (73.5)	24 (30.8)	
inhomogeneous	40 (26.5)	54 (69.2)	
Size of thyroid gland			<0.0001
normal	132 (87.4)	40 (51.3)	
increased	19 (12.6)	38 (48.7)	
decreased	0	0	
Margin of thyroid gland			<0.0001
smooth	134 (88.7)	48 (61.5)	
lobulated	17 (11.3)	30 (38.5)	
Degree of enhancement			<0.0001
iso-(normal)	145 (96)	58 (74.4)	
decreased	4 (2.7)	19 (24.4)	
increased	2 (1.3)	1 (1.2)	
Pattern of enhancement			<0.0001
homogeneous	131 (86.8)	31 (39.7)	
inhomogeneous	20 (13.2)	47 (60.3)	
CT classification			<0.0001
No DTD	85 (56.3)	4 (5.1)	
indeterminate	33 (21.9)	7 (9)	
suspicious for DTD	19 (12.6)	23 (29.5)	
DTD	14 (9.3)	44 (56.4)	

Note.—

* Data of HU are mean ± standard deviation.

Data presented in parentheses are percentage of each item. CT, computed tomography; DTD, diffuse thyroid disease; HU, Hounsfield Unit; NECT, non-enhanced CT; CECT, contrast-enhanced CT.

The mean (± SD) HU values of the thyroid parenchyma on both non-enhanced and contrast-enhanced CT images were significantly different between patients with NTP (107.9 ± 19.4 and 209.5 ± 36.3, respectively) and those with DTD (92.7 ± 25.3 and 192.9 ± 35.2, respectively). The ROC curve analysis revealed that the optimal parenchymal HU cut-off values for differentiating DTD from NTP were as follows: <103 HU (i.e., decreased attenuation) on non-enhanced CT images (Az = 0.716; 95% CI: 0.653, 0.773; *p* < 0.0001) and <205 HU on contrast-enhanced CT images (Az = 0.618; 95% CI: 0.553, 0.683; *p* = 0.002). The diagnostic accuracy for identifying DTD using a cut-off value of <103 HU on non-enhanced CT had a sensitivity of 75.6%, specificity of 63.6%, positive predictive value of 51.8%, and negative predictive value of 83.5%. On contrast-enhanced CT images, a cut-off value of <205 HU exhibited a diagnostic accuracy with a sensitivity of 67.9%, specificity of 56.3%, positive predictive value of 44.5%, and negative predictive value of 77.3%.

Comparisons of the CT diagnoses and histopathological results are summarized in Table 2. According to the linear-by-linear association test with a 2 × 4 contingency table, the incidence of DTD increased along with the CT category (*p* < 0.0001).

**Table 2:** Comparison of the computed tomography diagnoses and histopathological results in 229 patients.

Histopathology CT category	Hashimoto thyroiditis	Non-Hashimoto lymphocytic thyroiditis	Diffuse hyperplasia	Normal thyroid parenchyma
no DTD (n = 89)	1 (1.1)	3 (3.4)	0	85 (95.5)
indeterminate (n = 40)	1 (2.5)	6 (15)	0	33 (82.5)
suspicious for DTD (n = 42)	10 (23.8)	11 (26.2)	2 (4.8)	19 (45.2)
DTD (n = 58)	12 (20.7)	27 (46.6)	5 (8.6)	14 (24.1)

Note.—Data presented in parentheses are percentage of each item. CT, computed tomography; DTD, diffuse thyroid disease.

### Diagnostic accuracy of the CT features

Univariate logistic regression analyses of the CT features revealed that decreased attenuation, inhomogeneous attenuation, increased glandular size, a lobulated margin, inhomogeneous enhancement, and a higher CT category were independent predictors that were capable of distinguishing DTD from NTP. Multivariate logistic regression analyses of the CT features showed that the CT classification system utilizing the number of abnormal CT features was the only independent predictor associated with DTD (Table 3).

**Table 3:** Logistic regression analyses of the computed tomography findings for differentiating diffuse thyroid disease from normal thyroid parenchyma in 229 patients.

	Univariate Logistic Regression		Multivariate Logistic Regression	
Variables	Odds Ratio*	*P v*alue	Adjusted Odds Ratio*	*P* value
Age	0.98 (0.97, 1.01)	0.183	NA	
Degree of attenuation	6.50 (3.51, 12.06)	<0.0001	1.01 (0.34, 3.06)	0.982
Pattern of attenuation	6.24 (3.42, 11.39)	<0.0001	0.42 (0.11, 1.53)	0.998
Size	6.60 (3.43, 12.71)	<0.0001	1.13 (0.39, 3.28)	0.823
Margin	4.93 (2.49, 9.73)	<0.0001	0.74 (0.24, 2.41)	0.626
Degree of enhancement	11.88 (3.87, 36.41)	<0.0001	1.80 (0.45, 7.52)	0.406
Pattern of enhancement	9.93 (5.17, 19.09)	<0.0001	1.11 (0.35, 3.52)	0.855
CT classification	12.72 (7.96, 28.94)	<0.0001	24.35 (12.98, 41.56)	0.003
HU on NECT	0.97 (0.95, 0.98)	<0.0001	0.99 (0.97, 1.03)	0.832
HU on CECT	0.98 (0.97, 0.99)	0.011	1.00 (0.99, 1.02)	0.805

Note.—

* Numbers in parentheses are 95% confidence intervals.

NA = not available (values are not presented for factors that were not included or found to be insignificant in multivariate analysis). CT, computed tomography; HU, Hounsfield Unit; NECT, non-enhanced CT; CECT, contrast-enhanced CT.

**Fig 3:**
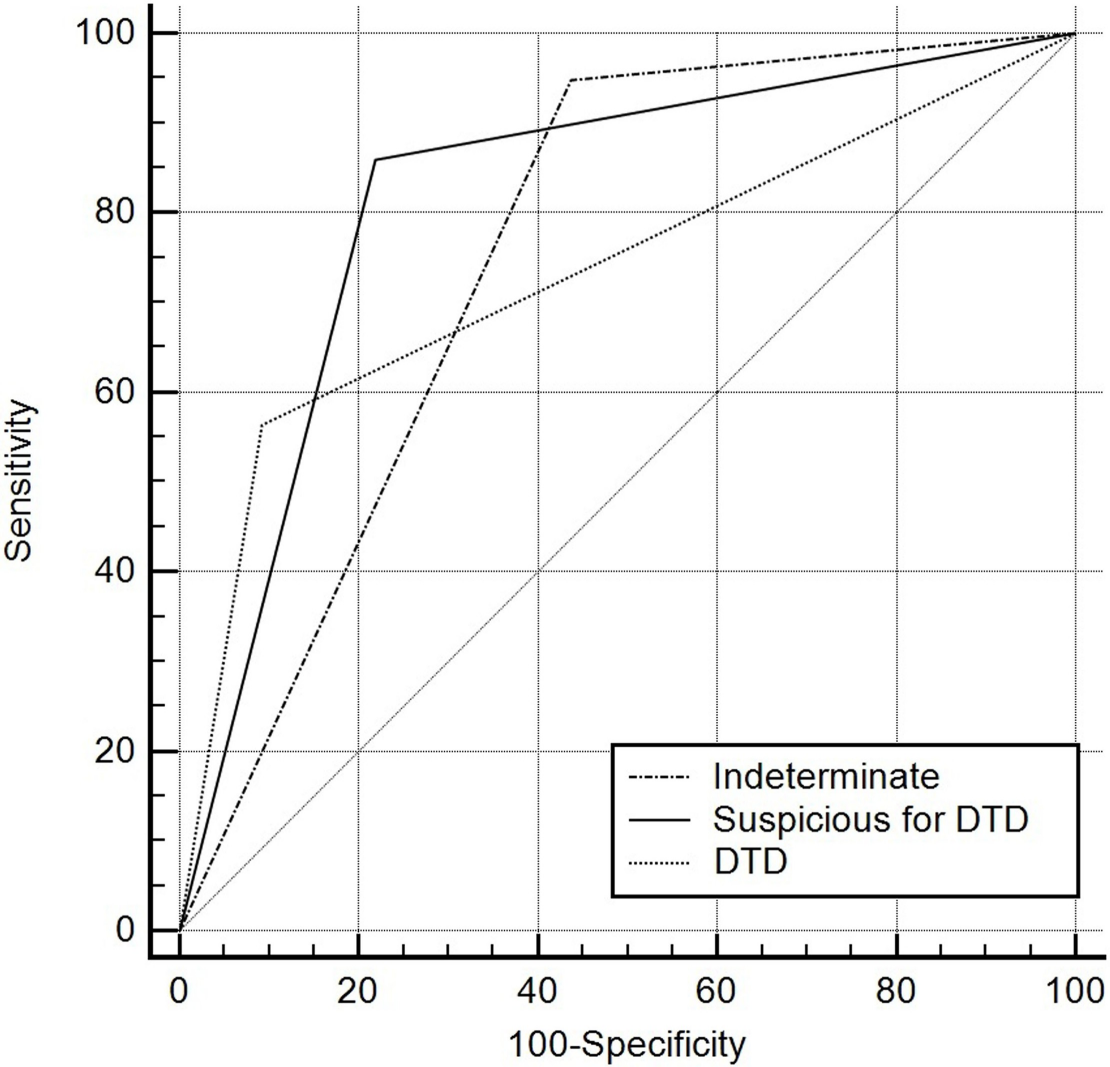
Comparisons of the receiver operating characteristic (ROC) analyses for the diagnostic accuracy of the cut-off computed tomography category to differentiate diffuse thyroid disease from normal thyroid parenchyma. Diagonal line = 50% of the area under the ROC curve; also refers to a hypothetical marker that has no discriminatory power for differentiating diffuse thyroid disease from normal thyroid parenchyma.

In addition, the ROC curve analyses showed that the cut-off “suspicious for DTD” category (i.e., ≥2 abnormal CT features) was the best predictor of DTD and had the largest area under the ROC curve (Az = 0.820; 95% CI: 0.764, 0.868), with a sensitivity of 85.9% and a specificity of 78.2% (Fig 3 and Table 4).

**Table 4:** Diagnostic accuracy of the computed tomography classification for differentiating diffuse thyroid disease from normal thyroid parenchyma in 229 patients.

Cut-off CT category	A_*z*_ value*	Sensitivity (%)	Specificity (%)	PPV (%)	NPV (%)	*P* value
Indeterminate	0.756 (0.695, 0.810)	94.9	56.3	52.9	95.5	<0.0001
Suspicious for DTD	0.820 (0.764, 0.868)	85.9	78.2	67.0	91.5	<0.0001
DTD	0.736 (0.674, 0.792)	56.4	90.7	75.9	80.1	<0.0001

Note.—

* Numbers in parentheses are 95% confidence intervals.

*A*_*z*_ means the largest area under the ROC curve. CT, computed tomography; DTD, diffuse thyroid disease; NPV, negative predictive value; PPV, positive predictive value.

### Analyses of the CT diagnoses according to institution

Of the five participating institutions, the degree and pattern of parenchymal attenuation, glandular size and margin, and pattern of parenchymal enhancement were significantly different between patients with DTD and those with NTP at three institutions (*p* values ranging from <0.0001 to 0.019). However, for one of the two remaining institutions, only the CT categories were significantly different between patients with DTD and patients with NTP, while the individual CT features were not significantly different between the groups. For the fifth institution, most of the individual CT features were significantly different between the DTD and NTP groups, except for the degree of parenchymal enhancement, which was recorded as normal in all cases.

The CT diagnosis for DTD at each institution is summarized in Table 5. Comparative analyses demonstrated that the CT diagnosis was significantly different between patients with DTD and those with NTP for all participating institutions (*p* < 0.05); moreover, we identified a positive linear correlation between the incidence of DTD and the CT category (*p* < 0.0001). The diagnostic accuracy of each reader at the participating institutions was also examined (Table 6). The diagnostic accuracy of each reader was significantly different (*p* < 0.0001). However, the relationship between readers’ experience and their diagnostic accuracy was not statistically significant (τ = -0.59, *p* = 0.318).

**Table 5:** Comparison of the computed tomography category for differentiating diffuse thyroid disease from normal thyroid parenchyma according to institution.

Institution	CT category	NTP	DTD	P value
A (n = 45)				0.001
	No DTD	12 (48)	1 (5)	
	indeterminate	5 (20)	2 (10)	
	suspicious for DTD	7 (28)	9 (45)	
	DTD	1 (4)	8 (40)	
B (n = 44)				<0.0001
	No DTD	26 (83.9)	2 (15.4)	
	indeterminate	3 (9.7)	0	
	suspicious for DTD	1 (3.2)	0	
	DTD	1 (3.2)	11 (84.6)	
C (n = 50)				0.012
	No DTD	15 (42.9)	1 (6.7)	
	indeterminate	10 (28.6)	4 (26.7)	
	suspicious for DTD	1 (2.9)	4 (26.7)	
	DTD	9 (25.7)	6 (40)	
D (n = 40)				<0.0001
	No DTD	9 (36)	0	
	indeterminate	9 (36)	0	
	suspicious for DTD	4 (16)	5 (33.3)	
	DTD	3 (12)	10 (66.7)	
E (n = 50)				<0.0001
	No DTD	23 (65.7)	0	
	indeterminate	6 (17.1)	1 (6.7)	
	suspicious for DTD	6 (17.1)	5 (33.3)	
	DTD	0	9 (60)	

Note.—Data presented in parentheses are percentage of each item. CT, computed tomography; NTP, normal thyroid parenchyma; DTD, diffuse thyroid disease.

**Table 6:** Diagnostic accuracy of computed tomography for differentiating diffuse thyroid disease from normal thyroid parenchyma according to institution (when a cut-off “suspicious for DTD” category was selected).

Institution	A_*z*_ value*	Sensitivity (%)	Specificity (%)	PPV (%)	NPV (%)	*P* value
A (n = 45)	0.831 (0.690, 0.926)	85	68	68	85	<0.0001
B (n = 44)	0.897 (0.768, 0.968)	84.6	83.9	68.8	92.9	<0.0001
C (n = 50)	0.710 (0.564, 0.829)	66.7	71.4	50	83.3	<0.0001
D (n = 40)	0.893 (0.755, 0.969)	100	72	68.2	100	<0.0001
E (n = 50)	0.954 (0.855, 0.993)	93.3	82.9	70	96.7	<0.0001

Note.—*A*_*z*_ means the largest area under the ROC curve.

*Numbers in parentheses are 95% confidence intervals. NPV, negative predictive value; PPV, positive predictive value.

## Discussion

In the current study, we found that the CT diagnosis was helpful for detecting incidental DTD. The presence of two or more abnormal CT features (i.e., “suspicious for DTD” or “DTD” category) had the highest Az value, indicating that this classification had the highest diagnostic accuracy, which is consistent with the published literature [6]. The diagnostic values identified here were similar to those reported in previous studies that diagnosed DTD using ultrasonography or CT, but the negative predictive value of the current study was higher than the previously reported values [5–8]. However, the recent study showed that the presence of three or more abnormal CT features had the greatest diagnostic accuracy but lower sensitivity [7]. Regardless, to our knowledge, this is the first multicenter study to demonstrate the feasibility of using CT to evaluate and diagnose DTD by revealing a correlation between the CT findings and the histopathological results.

According to our results, the CT category was significantly different between patients with DTD and patients with NTP at all participating institutions, and a positive linear correlation was identified between the incidence of DTD and the CT category. In addition, the diagnostic accuracy of each reader was variable. Unlike the CT category, the individual CT features showed different frequencies between patients with DTD and those with NTP at each institution. However, specific CT features of DTD were not found. Based on these results, we suggest that the CT category and a combination of individual CT features for DTD may be a reliable marker for distinguishing DTD from NTP. However, there was difference in CT modality, study patients, and sample size between participating institutions. Thus, this may have affected the results of the current study. The individual readers’ experience in neck CT interpretation may be associated with diagnostic performance in detection of DTD. One study reported that there was no significant difference between readers’ experience and diagnostic accuracy [9]. In contrast, another study stated that diagnostic accuracy increased with experience of the radiologist [11]. In our study, no significant association was found between the readers’ experience and diagnostic accuracy. Therefore, to clarify these issues, further studies using cross-review are required.

In the present study, the DTD and NTP groups were significantly different in terms of the mean HU values on both non-enhanced CT and contrast-enhanced CT images. The optimal cut-off HU value on non-enhanced CT images was determined to be 103 HU, implying that parenchymal attenuation <103 HU likely indicates DTD. In a previous study, the cut-off HU value for diagnosing DTD on non-enhanced CT images was 100 HU, which is similar to the value reported here [7]. However, unlike the previous study, we also calculated the optimal cut-off HU value for the degree of parenchymal enhancement on contrast-enhanced CT images, which was 205 HU. Nevertheless, it should be noted that we did not use the more-accurate volumetric approach to measure the HU value; rather, we measured the HU value in the thyroid gland by applying circular regions of interest in the picture-archiving and communication system. Therefore, additional studies may be required to determine the optimal cut-off HU value for diagnosing DTD.

There are several limitations in this study. First, there was unavoidable selection bias because the data from all patients were evaluated retrospectively. Furthermore, all of the study patients underwent thyroid surgery. Although this factor was necessary for correlating the CT findings with the histopathological results as a reference standard, sampling bias might have occurred. Second, we did not include clinical and serological data for comparison. Third, subject determination of individual CT features was performed at each institution. Fourth, retrospective CT image analyses were performed by five radiologists with different experience. Thus, the potential of inter-observer variability should be considered. Lastly, we did not investigate the effects that the use of different CT scanners and imaging protocols at the participating institutions might have had on our findings. Although the data were obtained from daily clinical practice, these factors may have affected the CT analyses in the current study.

## Conclusions

Our study demonstrates that the CT diagnosis is helpful for differentiating DTD from NTP regardless of the experience of the investigators. In particular, when two or more abnormal CT features are observed, the possibility of DTD should be considered.

## Supporting information

S1 File Dataset. (XLS)
